# The Use of Imaging to Quantify the Impact of Seed Aging on Lettuce Seed Germination and Seedling Vigor

**DOI:** 10.3390/s24134235

**Published:** 2024-06-29

**Authors:** Mark Iradukunda, Marc W. van Iersel, Lynne Seymour, Guoyu Lu, Rhuanito Soranz Ferrarezi

**Affiliations:** 1 Department of Horticulture, University of Georgia, Athens, GA 30602, USA; mark.iradukunda@uga.edu (M.I.); mvanier@uga.edu (M.W.v.I.); 2 Department of Statistics, University of Georgia, Athens, GA 30602, USA; seymour@uga.edu; 3 College of Engineering, University of Georgia, Athens, GA 30602, USA; guoyu.lu@uga.edu

**Keywords:** canopy size, vigor, image analysis, *Lactuca sativa*, plant modeling, seed deterioration

## Abstract

The decline in seed quality over time due to natural aging or mishandling requires assessing seed vigor for resilience in adverse conditions. Accelerated aging (AA) methods simulate seed deterioration by subjecting seeds to high temperatures and humidity. Saturated salt accelerated aging (SSAA) is an AA method adopted for small seeds like lettuce (*Lactuca sativa*). In this study, we subjected seeds of two lettuce cultivars (‘Muir’ and ‘Bauer’) to SSAA by sealing them in a box containing 40 g/100 mL of a sodium chloride (NaCl) solution in a dark growth chamber at 41 °C for 24, 48, and 72 h with a control. We monitored their vigor using embedded computer cameras, tracking the projected canopy size (PCS) daily from sowing to harvest. The cultivar ‘Muir’ exhibited consistent PCS values across the treatments, while ‘Bauer’ showed PCS variations, with notable declines after prolonged aging. The germination rates dropped significantly after 48 and 72 h of SSAA. A nonlinear regression model revealed a strong relationship between PCS and shoot dry weight across harvests and cultivars (*R*^2^ = 0.93, *RMSE* = 0.15, *p* < 0.001). The research found that the projected canopy size and shoot dry weight increased over time with significant differences in treatments for the cultivar ‘Bauer’ but not for ‘Muir,’ with the canopy size being a strong predictor of dry weight and no significant impact from the SSAA treatments. This study highlights cultivar-specific responses to aging and demonstrates the efficacy of our imaging tool in predicting lettuce dry weight despite treatment variations. Understanding how aging affects different lettuce varieties is crucial for seed management and crop sustainability.

## 1. Introduction

Seed vigor decreases over time because seed-stored metabolites and cellular integrity deteriorate as time passes, leading to ‘seed aging’ [1]. Seed aging starts with DNA and protein oxidative damage by oxygen-reactive species [2,3]. This negatively affects the seed vigor, where repairs needed to drive the early germination stage are compromised. Aging and deterioration are influenced by the moisture content and temperature fluctuations, but results are different across plant species. This knowledge helps us to understand the required conditions for long-term storage of plants. For instance, seed storage at low temperatures and humidity can keep seeds vigorous for over 50 years [4]. This is where seed vigor can be quantified and compared amongst seed lots of different plant species. If the seed germinates, the seed is considered viable. The seed lot where a high percentage of seeds sprout into healthy plants in various conditions is deemed to have high vigor [2,3,5].

As some seeds can last for decades, scientists have developed ways to speed up seed aging by using accelerated aging (AA) to assess their quality. In AA, also known as artificial aging/deterioration, seeds are exposed to high moisture and temperatures to speed up aging. Many research papers separate accelerated aging (high temperatures; 45 °C) and controlled deterioration (high temperature and humidity), but the goal is the same: to age the seed. The standard procedure requires around 40 °C and 20% moisture content [6]. After that, the seeds are germinated to evaluate their vigor and long-term storability. In the same way, seeds that have been stored for a long time can be assessed to determine if they can be stored longer or if they should be discarded. The challenge with AA is that small seeds, like vegetable seeds, absorb water too fast, which makes it hard to make accurate seed lot evaluations based on this method. For that reason, scientists developed saturated salt accelerated aging (SSAA) as more beneficial for small seeds like lettuce [7]. In SSAA, salt is added to reduce water uptake, decreasing variations in artificially aged seeds and making the assessment more uniform across seed types and sizes [7]. The aged seeds are treated by either method, and their quality is evaluated based on their germination performance. However, the initial germination test is usually binary (germinated or not) and does not provide information on the seedling vigor or how the plant will likely perform in the future [8]. For that reason, subjecting the seeds to less-than-ideal conditions and evaluating their performance can provide more insights into seed lot potential yields and storability [6,8,9]. 

Conventional techniques in assessing seed quality and seedling vigor, such as cold tests, electrical conductivity tests, and tetrazolium tests, are often hampered by several drawbacks. They typically involve time-consuming processes, demand significant manual effort, and may not accurately predict field performance [1,10]. Additionally, these methods often require large quantities of seeds and are less capable of detecting minor differences in seed quality [11]. In contrast, image-based techniques offer a non-destructive, efficient, and precise alternative for assessing seed quality and vigor. By utilizing advanced imaging technologies and machine learning, these methods can analyze the characteristics of seeds and seedlings with greater sensitivity and provide real-time evaluation capabilities [3,12]. Our study assessed the seedling performance by recording PCS from sowing to the final harvest using non-destructive embedded computer cameras installed in a walk-in growth chamber. We direct the reader to our other publication to learn about our technique in detail and its uniqueness [13].

We employed the SSAA method, involving high temperature and humidity, to simulate the aging process of lettuce seeds over varying durations. Subsequently, we assessed the impact of this artificial aging on the germination rate, uniformity, and seedling vigor over time. We hypothesize that seeds subjected to artificial aging would show diminished germination rates and reduced canopy size and biomass compared to the control group. Furthermore, we expected this impact to intensify with prolonged exposure to aging.

## 2. Materials and Methods

### 2.1. Location and Environmental Conditions

The study took place at the University of Georgia (College of Agricultural and Environmental Sciences, Department of Horticulture, Horticultural Physiology and Controlled Environment Agriculture Laboratories) in Athens, GA (latitude 33°57′26.676″ N, longitude 83°22′36.48″ W). The plants were cultivated in a walk-in growth chamber. Conditions were kept at an air temperature of 25 °C, relative humidity of 70%, a vapor pressure deficit of 0.91 kPa, a carbon dioxide level of 800 mg/L, and a light intensity of 250 µmol·m^2^·s^−1^ for a 16-h photoperiod (from midnight to 4 p.m.). This resulted in a daily light integral of 14.4 mol·m^−2^·d^−1^. For irrigation, the plants were watered daily with a 15N-2.2P-12.5K fertilizer solution containing 100 mg/L of nitrogen (15-5-15 Ca-Mg Professional LX; J.R. Peters, Allentown, PA, USA) through an automated ebb-and-flow subirrigation system that ran for 5 min each day.

### 2.2. Seed Aging Procedure

We selected two green-colored lettuce cultivars (‘Bauer’ and ‘Muir’; Jonny’s Selected Seeds, Winslow, ME, USA). Following the standardized procedure described in [14], we subjected the lettuce seeds to an accelerated aging process. Specifically, the SSAA protocol required the exposure of seeds to a 40 g/100 mL granular sodium chloride (NaCl; Thermo Fisher Scientific, Waltham, MA, USA) solution at 41 °C. Seeds from each cultivar were divided into four groups, each subjected to different aging durations: 0 (control), 24, 48, and 72 h. Commercial aging boxes and custom-made boxes made with a few materials can be used. The most important aspect is that the seeds cannot come into contact with the solution, and the box must be completely sealed. We used commercial germination boxes with outside dimensions of approximately 24.13 × 16.03 × 3.81 cm (Acrylic Container 6 × 9; Hoffman & Company, Corvallis, OR, USA) and a wire mesh (60 Stainless Steel Mesh; Ovsor Mesh & Wire Cloth Store, China). We used small wood panels to make a rectangular structure that fitted inside the box and fixed the wire mesh at the top. In the center of the structure, we put a small piece of wood to make two sections for the two cultivars. 

**Figure 1:**
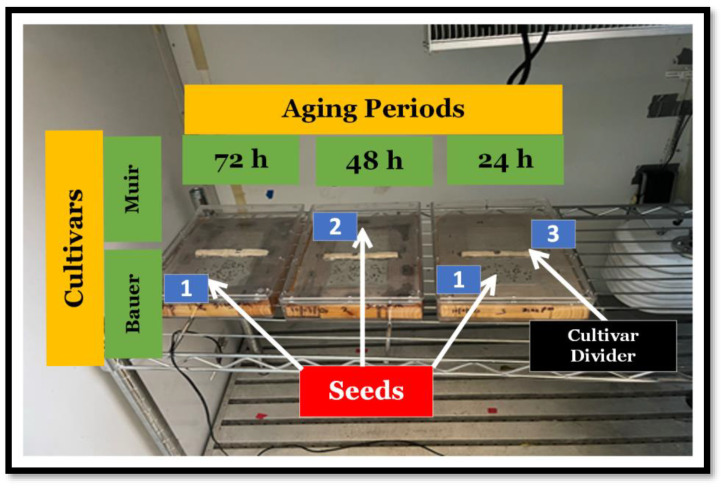
Visual representation of seed treatment arrangements (excluding the control, 0 h) placed inside the growth chamber. Each sealed box represents the time they were aged for, starting with 72 h. The wood panels support the wire mesh, leaving space between the solution and the seeds. (1) Dark-colored seeds belonging to the cultivar ‘Bauer’. (2) Light-colored seeds belonging to the cultivar ‘Muir’. (3) Cultivar divider. The piece of wood that separates the seeds belonging to different cultivars.

After properly making the box and adding the prepared aging solution, a single layer of uncoated seeds from each group was evenly spread on the wire mesh, and the box was sealed. The sealed containers served as chambers for the aging treatment. The seeds aging for 72 h were placed in the growth chamber first, the next day those aging for 48 h, and on the last day, those aging for 24 h. All seeds of the two cultivars were kept in the same germination box to ensure that all seeds of both cultivars were handled in the same manner (Figure 1).

Subsequently, the containers were positioned within the same growth chamber (CMP3244; Conviron-Controlled Environments Limited, Winnipeg, MB, Canada) maintained at a constant temperature of 41 °C, and they were kept in the dark, as previously described by [15]. The growth chamber functioned solely as a temperature control environment, ensuring the maintenance of the specified conditions for the seed aging process.

### 2.3. Experimental Setup, Image Acquisition, and Image Analysis

After the aging treatments, the aged seeds were sown normally and placed in a walk-in chamber. One hundred twenty-eight seeds were used, with four seeds per treatment (four treatments/replication) and sixty-four seeds for each cultivar (four replications per cultivar). Embedded cameras were used to monitor their growth from sowing to final harvest (28 days). Useful data, including projected canopy size, germination rate, and shoot dry weight, were collected. The plant growth management, imaging, and data analysis methods we used are described in our previous study [13]. Because the space between the cameras and the plants was small, we regularly moved the plants around to ensure they were in the camera’s view to allow us to obtain proper images and for the accurate projection of the canopy size. We collected dry weight data thrice (12, 21, and 28 days after sowing). In the data analysis, we compared four treatments and two cultivar effects. 

## 3. Results

### 3.1. Projected Canopy Size over Time

**Figure 2:**
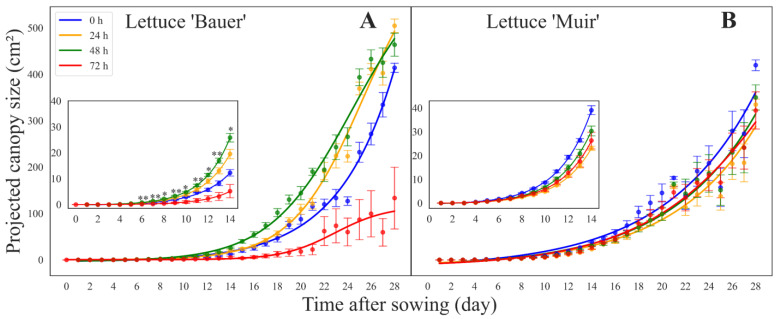
Curves showing the relationship between the projected canopy size (PCS) of the lettuce (*Lactuca sativa*) seedlings of each saturated salt accelerated aging (SSAA) period of the cultivars (**A**) ‘Bauer’ and (**B**) ‘Muir’ obtained by embedded computers and time (days after sowing). PCS data were collected every day from sowing to 14 days. Each data point and the error bars indicate the mean and standard deviation of 16 seedlings for each day in each treatment/cultivar. Inserts display PCS for the seedling stage (initial 14 days after sowing). * and ** indicate significant differences between the cultivars using Tukey’s HSD at α ≤ 0.05 and <0.01, respectively.

The PCS increased over time in all treatments and two lettuce cultivars (Figure 2). Initially, no germination was observed, but around day five, PCS slightly increased, followed by exponential growth afterward. Contrary to our hypothesis, the seeds of the cultivar ‘Bauer’ treated with SSAA for 48 h showed a greater size and fast growth throughout the germination period, followed by those treated for 24, 0 (control), and 72 h. We observed significant differences in PCS on days 6, 7, 8, 9, 10, 11, 12, 13, and 14 (Figure 2A).

No statistically significant differences in PCS were observed in the SSAA treatments for the cultivar ‘Muir’ (Figure 2B). In both cultivars, the initial performances among the treatments in the first 14 days after sowing continued to be seen throughout the growth cycle.

### 3.2. Germination Curves

**Figure 3:**
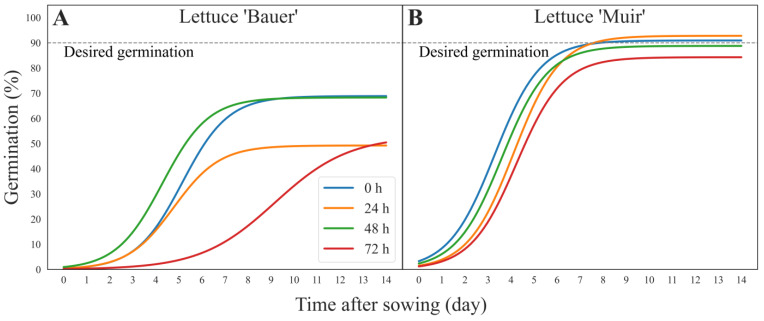
Sigmoidal curves showing the germination percent over time colored according to the saturated salt accelerated aging (SSAA) periods in the lettuce (*Lactuca sativa*) cultivars (**A**) ‘Bauer’ and (**B**) ‘Muir’. Germination percent calculated from data obtained by the embedded computers. The gray dashed line shows the desired germination (90%), and where the curve does not reach, the line means poor germination.

Significant variations were observed in the germination percentages among the lettuce cultivars (Figure 3). None of the SSAA treatments in the cultivar ‘Bauer’ reached the 90% desired germination (Figure 3A). As expected, the seeds treated with SSAA for 72 h exhibited the slowest and worst germination. SSAA for 0 h (control) and 48 h showed the best germination.

For the cultivar ‘Muir’, SSAA for 24 h and 0 h (control) had a faster germination, while SSAA for 72 h had a slower germination (Figure 3B). It took about 8 days for SSAA (24 h) and the control (0 h) to reach 90% germination, while the 48- and 72-h treatments did not reach the desired germination.

### 3.3. Shoot Dry Weight

**Figure 4:**
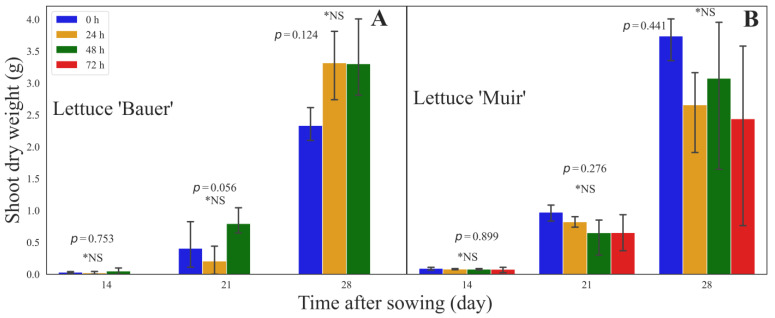
Shoot dry weight collected at 14, 21, and 28 days after sowing of the lettuce (*Lactuca sativa*) cultivars (**A**) ‘Bauer’ and (**B**) ‘Muir’. Each bar and the error bars indicate the mean and standard deviation of 16 seedlings in each saturated salt accelerated aging (SSAA) period. There were four SSAA periods in total, but the cultivar ‘Bauer’ seeds treated for 72 h (A) did not survive and the treatment was removed from the analysis (hence, only three treatment periods). * NS in each harvest denotes a non-significant difference, and values followed by the same letter are not significantly different according to Tukey’s HSD at α = 0.05.

For the cultivar ‘Bauer’, no significant treatment variations were observed in the shoot dry weight on day 14 (*p* = 0.753), day 21 (*p* = 0.056), and day 28 (*p* = 0.124) after sowing (Figure 4A). The average weights for SSAA periods 0 (control), 24, and 48 h were 0.031, 0.020, and 0.049 g on day 14; 0.407, 0.206, and 0.795 g on day 21; and 2.332, 3.318, and 3.302 g on day 28, respectively (Figure 4A). Similarly, there were no significant differences in the shoot dry weight between the treatments for the cultivar ‘Muir’ (*p* = 0.899) on day 14 after sowing and day 21 (*p* = 0.276), and the final shoot dry weight on day 28 (*p* = 0.441) (Figure 4B). The average weights for the SSAA periods 0 (control), 24, and 48 h were 0.085, 0.075, 0.072, and 0.072 g on day 14; 0.911, 0.770, 0.610, and 0.610 g on day 21; and 3.502, 2.490, 2.880, and 2.285 g on day 28, respectively (Figure 4B).

**Figure 5:**
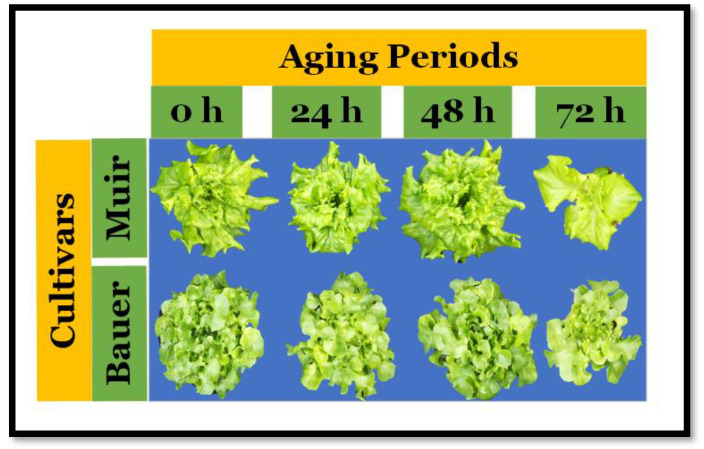
Images of lettuce (*Lactuca sativa*) plants arranged by cultivar and different saturated salt accelerated aging periods at 28 days after sowing. (0 h): The control seeds were not exposed to the aging procedure. Images shown are for visual purposes only and do not represent any statistical analysis result.

Statistical analysis both of PCS and the dry weight showed no significant differences. Looking at the mature plant images, there were no visible differences except for the plants that resulted from seeds aged for 72 h (Figure 5). These plants showed a reduced size and pale leaves and did not properly reach maturity as the other plants (head formation). However, these variations were not significant in the dry weight of the cultivar ‘Muir’, and the cultivar ‘Bauer’ did not have enough plants to allow multiple comparison tests.

### 3.4. PCS and Shoot Dry Weight

**Figure 6:**
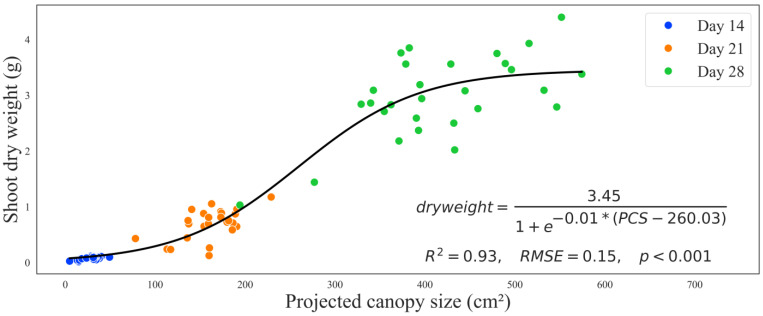
The relationship between the projected canopy size (PCS) and the corresponding shoot dry weight colored by days of harvest (to collect the shoot dry weight). The black solid line shows a sigmoidal regression with an equation, regression summaries of the coefficient of determination (R^2^), root-mean-square error (RMSE), and *p*-value (*p*).

Combining all canopy size data with all three harvests revealed that the canopy size was a good predictor of the dry weight (*t* = 26.735, *p* < 0.001). While the cultivar had a significant effect at (*t* = 2.700, *p* = 0.009), the SSAA treatments did not show a significant effect at (*t* = −0.625, *p* = 0.534). A sigmoidal regression model (generalized for all three harvests and two cultivars) showed an S-shaped pattern between PCS and the shoot dry weight (*R^2^* = 0.93, *RMSE* = 0.15, *p* < 0.001) (Figure 6). From the regression equation, the maximum dry weight was 3.45 g, with a growth rate of 0.01 g/cm^2^. To reach half of the maximum growth rate, a PCS of 260.03 cm^2^ was required.

## 4. Discussion

Exposing seeds to high temperatures (>30 °C) is known to hamper germination by promoting the hardening of the endosperm cell wall [16] and making it difficult for the radicle to emerge. In addition to mechanically hindering germination, elevated temperatures lead to the damage of DNA material [5] that facilitates early germination at the molecular level. For example, a gene required to maintain seed viability in *Arabidopsis* (AtLIG6) must be repaired for germination. That explains why the lettuce seeds treated with SSAA for 72 h performed poorly in PCS (Figure 2A) and germination percent (Figure 3A). Furthermore, the majority of the ‘Bauer’ seeds treated with SSAA for 72 h did not survive, leading to no recorded shoot dry weight (Figure 4A), indicating irreparable damage. The reduced seedling and subsequent plant performance because of seed aging aligns with a similar study that aged lettuce seeds for 72 h [15]. In that study, SSAA seemed to make lettuce seeds vulnerable to diseases and the accumulation of toxins, which greatly reduced vigor [15]. In another standard seed test, pepper (*Capsicum annuum* L.) seedlings were evaluated based on the germination speed and uniformity, and seed aging was found to reduce seed and seedling quality [17]. A similar effect of aging seeds on their storage potential was also observed in cucurbits [6]. Despite the generally held assumptions regarding the adverse effects of aging on PCS and germination, these expectations did not align with the outcomes observed in the cultivar ‘Bauer’. Surprisingly, the SSAA treatment for 48 h exhibited higher PCS (Figure 2A) and germination (Figure 3A) in the cultivar ‘Bauer’, despite the absence of significant differences in the shoot dry weight (Figure 4A). This unexpected anomaly remains unexplained, with no parallel findings reported in current studies. However, the anomaly appears to be specific to this particular cultivar. While these results do not undermine our overall conclusions, they underscore the importance of subjecting seeds to standard seed aging tests lasting 72 h to ensure consistent outcomes comparable to those of other studies [15].

‘Muir’ is claimed to be a heat-tolerant cultivar by seed companies. That claim was verified when ‘Muir’ did well among other cultivars [18]. Our results suggest that the cultivar ‘Muir’ is excellent in growing systems, and its seeds are resistant to aging treatments (SSAA). The cultivar’s resilience was evident as there were no notable variations in PCS due to aging treatments (Figure 2B). The germination of the ‘Muir’ cultivar was adversely affected only after 48 h of exposure to elevated humidity and temperature (Figure 3B), highlighting its robustness. However, after 48 and 72 h, germination rates did not exceed 90%, with the latter period exhibiting the lowest performance. In addition, we saw a visible suboptimal performance in color and head formation in mature plants from seeds treated for 72 h (Figure 5). It has also been suggested that the differences in germination and vigor among seeds can be influenced by the seedcoat color, where dark seeds performed better [15]. In our study, the cultivar ‘Muir’, a white-coated seed, performed better than the dark-coated cultivar ‘Bauer’. Though controlled deterioration tests such as SSAA are useful to understand the performance of seeds, small seeds like lettuce are challenging because a small seed’s moisture content is difficult to monitor due to its small size [17]. In this study, the seed response to deterioration and exposure time was significantly influenced by seed moisture content [17]. 

A sigmoidal regression model demonstrated the nonlinear relationship between PCS and the shoot dry weight across all harvests and cultivars (*R*^2^ = 0.93, *RMSE* = 0.15, *p* < 0.001) (Figure 6). The sigmoidal curve demonstrates that the shoot dry weight rises with the increase in PCS in an S-shaped pattern. A greater surface area of the plant (leaf exposure) has been associated with more light interception for photosynthesis and an increase in yield [19]. Comparable results in predicting dry weight from PCS have also been reported in lettuce [20]. The regression line increases exponentially, showing that, as the plant grows after germination, the shoot dry weight will increase faster than PCS due to the leaf number and thickness [21]. The line then plateaus as the plants mature, forming an S-shaped (sigmoidal) curve, indicating that plant resources are used elsewhere besides vegetative growth [22]. One considerable factor that hinders the accuracy of our predictions is that mature plants have more leaves hidden, contributing to the greater variability at day 28. Similar challenges were observed in predicting lettuce growth [23].

## 5. Conclusions

These observed patterns suggest that SSAA treatments affect germination and early growth differently in lettuce cultivars. Notable exceptions include the unexpectedly positive response of ‘Bauer’ to SSAA for 48 h, contrasting with the hypothesized outcomes. SSAA for 72 h damaged seed metabolites, and those effects were seen in the initial germination and final harvest. Most ‘Bauer’ seeds treated for over 72 h did not survive, indicating that this cultivar has a lower vigor than ‘Muir’. This means that seed lots with a poor initial seed vigor will be more affected by aging and other harsh conditions, which is expected. Additionally, the lack of significant differences in the PCS of ‘Muir’ shows tolerance to aging stress in seeds and plants of that cultivar. The plants that performed well at the beginning continued to do so until the final harvest. The fact that early growth behaviors among treatments remained the same for PCS and dry weight highlights the potential of image analysis at the seedling stage to predict future plant performance. The strong correlation between PCS and dry weight proves that a non-destructive image analysis tool can be incorporated in controlled environment agriculture, especially in vertical farms, to provide useful information daily on plant behavior. 

This study emphasizes the importance of seed aging techniques in seed potential evaluation and how they can help to validate new tools. However, it is important to follow the procedures correctly and understand the kinds of crops one is dealing with. That is because stress responses are plant species-specific, and our tool dealt with lettuce, which has a unique growth. Seed tests continue to be improved to fit specific needs, and we hope that further studies will help to address small seed moisture content issues and their impact on germination and seedling vigor. As for technology, our largest constraint was how long we could run the experiment for before the plants grew out of the camera’s view due to shelf sizes. Great improvements in image analysis have been made, but there is still an issue with how to segment plants when they start touching each other. Similarly, some leaves are hidden as the plants grow, leading to inaccuracies in predicting the canopy size based on two-dimensional imaging. If further work could solve the inaccuracies in predicting canopy size, it would be possible to predict the shoot dry weight and other growth attributes more confidently.

## Data Availability

Dataset available upon request from the authors.

## References

[1] Xia Y., Xu Y., Li J., Zhang C., Fan S. (2019). Recent advances in emerging techniques for non-destructive detection of seed viability: A review. Artif. Intell. Agric..

[2] Rajjou L., Duval M., Gallardo K., Catusse J., Bally J., Job C., Job D. (2012). Seed germination and vigor. Annu. Rev. Plant Biol..

[3] Reed R.C., Bradford K.J., Khanday I. (2022). Seed germination and vigor: Ensuring crop sustainability in a changing climate. Heredity.

[4] Solberg S.Ø., Yndgaard F., Andreasen C., von Bothmer R., Loskutov I.G., Asdal Å. (2020). Long-term storage and longevity of orthodox seeds: A Systematic review. Front. Plant Sci..

[5] Snider J.L., Pilon C., Virk G., Chastain D.R., Kaur G., Reddy K.R., Oosterhuis D.M. (2020). Seed Characteristics and Seedling Vigor. Cotton Seed and Seedlings.

[6] Demir I., Mavi K. (2008). Controlled deterioration and accelerated aging tests to estimate the relative storage potential of cucurbit seed lots. HortScience.

[7] Marcos-Filho J. (2015). Seed vigor testing: An overview of the past, present and future perspective. Sci. Agric..

[8] Delouche J.C., Baskin C.C. (2021). Accelerated aging techniques for predicting the relative storability of seed lots. Seed Technol. Pap..

[9] Bennett M.A. (2004). Saturated Salt accelerated aging (SSAA) and other vigor tests for vegetable seeds. Seed Technol..

[10] Zhang J., Fang W., Xu C., Xiong A., Zhang M., Goebel R., Bo G. (2023). Current optical sensing applications in seeds vigor determination. Agronomy.

[11] Chen C., Bai M., Wang T., Zhang W., Yu H., Pang T., Wu J., Li Z., Wang X. (2024). An RGB image dataset for seed germination prediction and vigor detection-maize. Front Plant Sci..

[12] de Medeiros A.D., Capobiango N.P., da Silva J.M., da Silva L.J., da Silva C.B., dos Santos Dias D.C.F. (2020). Interactive machine learning for soybean seed and seedling quality classification. Sci. Rep..

[13] Iradukunda M., van Iersel M.W., Seymour L., Lu G., Ferrarezi R.S. (2024). Low-cost imaging to quantify germination rate and seedling vigor across lettuce cultivars. Sensors.

[14] Mcdonald M.B. (1997). The saturated salt accelerated aging test of pansy and impatiens seeds. Seed Technol..

[15] Peñaloza P., Ramirez-Rosales G., Mcdonald M.B., Bennett M.A. (2005). Lettuce (*Lactuca sativa* L.) seed quality evaluation using seed physical attributes, saturated salt accelerated aging and the seed vigour imaging system. Electron. J. Biotechnol..

[16] Park J.I., Cho D.M., Oh J.H., Cho J.S., Kang N.J. (2022). Improvement of germinability of lettuce seeds with drum-priming under high-temperature conditions. Hortic. Environ. Biotechnol..

[17] Basak O., Demir I., Mavi K., Matthews S. (2006). Controlled deterioration test for predicting seedling emergence and longevity of pepper (*Capsicum annuum* L.) seed lots. Seed Sci. Technol..

[18] Joukhadar I., Tonnessen B., Coon D., Walker S. (2023). Performance of heat-tolerant lettuce cultivars in southern New Mexico in 2020—21. HortTechnology.

[19] Christensen S., Goudriaan J. (1993). Deriving light interception and biomass from spectral reflectance ratio. Remote Sens. Environ..

[20] Kim C., van Iersel M.W. (2022). Morphological and physiological screening to predict lettuce biomass production in controlled environment agriculture. Remote Sens..

[21] Hoshino R., Yoshida Y., Tsukaya H. (2019). Multiple steps of leaf thickening during sun-leaf formation in Arabidopsis. Plant J..

[22] Weraduwage S.M., Chen J., Anozie F.C., Morales A., Weise S.E., Sharkey T.D. (2015). The relationship between leaf area growth and biomass accumulation in *Arabidopsis thaliana*. Front Plant Sci..

[23] Kim M.H., Choi E.G., Baek G.Y., Kim C.H., Jink B.O., Moon B.E., Kim D.E., Kim H.T. (2013). Lettuce growth prediction in plant factory using image processing technology. IFAC Proc. Vol..

